# Enhanced Cycling Stability of LiCu_x_Mn_1.95−x_Si_0.05_O_4_ Cathode Material Obtained by Solid-State Method

**DOI:** 10.3390/ma11081302

**Published:** 2018-07-27

**Authors:** Hongyuan Zhao, Fang Li, Xiuzhi Bai, Tingting Wu, Zhankui Wang, Yongfeng Li, Jianxiu Su

**Affiliations:** 1 School of Mechanical & Electrical Engineering, Henan Institute of Science and Technology, Xinxiang 453003, China; wtingtingwu@163.com (T.W.); luckywzk@126.com (Z.W.); yongfengli121@outlook.com (Y.L.); 2 Research Branch of Advanced Materials & Green Energy, Henan Institute of Science and Technology, Xinxiang 453003, China; lifang@hist.edu.cn; 3 School of Chemistry and Chemical Engineering, Henan Institute of Science and Technology, Xinxiang 453003, China; amibai@126.com

**Keywords:** lithium-ion batteries, cathode material, LiMn_2_O_4_, Cu-Si co-doping, cycling stability

## Abstract

The LiCu_x_Mn_1.95−x_Si_0.05_O_4_ (x = 0, 0.02, 0.05, 0.08) samples have been obtained by a simple solid-state method. XRD and SEM characterization results indicate that the Cu-Si co-doped spinels retain the inherent structure of LiMn_2_O_4_ and possess uniform particle size distribution. Electrochemical tests show that the optimal Cu-doping amount produces an obvious improvement effect on the cycling stability of LiMn_1.95_Si_0.05_O_4_. When cycled at 0.5 C, the optimal LiCu_0.05_Mn_1.90_Si_0.05_O_4_ sample exhibits an initial capacity of 127.3 mAh g^−1^ with excellent retention of 95.7% after 200 cycles. Moreover, when the cycling rate climbs to 10 C, the LiCu_0.05_Mn_1.90_Si_0.05_O_4_ sample exhibits 82.3 mAh g^−1^ with satisfactory cycling performance. In particular, when cycled at 55 °C, this co-doped sample can show an outstanding retention of 94.0% after 100 cycles, whiles the LiMn_1.95_Si_0.05_O_4_ only exhibits low retention of 79.1%. Such impressive performance shows that the addition of copper ions in the Si-doped spinel effectively remedy the shortcomings of the single Si-doping strategy and the Cu-Si co-doped spinel can show excellent cycling stability.

## 1. Introduction

Lithium-ion batteries have been applied extensively in a lot of power supply fields, like in pure electrical vehicles (EVs), unmanned aerial vehicles and smartphones. As one important part of lithium-ion batteries, cathode materials have played a crucial role in terms of electrochemical performance [1,2,3,4,5,6,7]. Among the existing cathode materials, LiMn_2_O_4_ possesses major advantages and great potential for the large-scale commercial application due to the mature production technology, cheap production costs and non-pollution characteristics [8,9,10]. It is important to note, however, that this material shows poor cycling stability and elevated-temperature performance, which produces a serious negative effect on promoting the large-scale commercial application. These unsatisfactory deficiencies are mainly caused by Jahn-Teller distortion and manganese dissolution [11,12,13,14].

According to the existing literatures [15,16,17,18], the body-doping strategy can improve the cycling stability to some degree by introducing other cations in the spinel structure. The common doping ions mainly include the monovalent ion (Li^+^) [19,20], divalent ions (Mg^2+^, Zn^2+^, Cu^2+^, etc.) [21,22,23,24], and trivalent ions (Al^3+^, Co^3+^, Cr^3+^, etc.) [25,26,27,28]. The research results have established that doping the trivalent manganese ions with these low valence cations can markedly improve the cycling life. However, introducing these low valence cations usually produces certain negative effects on the reversible capacity due to the decrease of Mn^3+^ ions. Considering this, doping the manganese ions with tetravalent cations has been developed to improve the electrochemical performance of LiMn_2_O_4_ because this strategy can avoid the decrease of trivalent manganese ions and reversible capacity loss of LiMn_2_O_4_ and provide the more expanded and stable MnO_6_ octahedra, which is conducive to the diffusion of lithium ions [29,30,31]. In the previous work [32], the Si-doped LiMn_2_O_4_ samples have been obtained by solid-state method. When cycled at 0.5 C, the optimal sample can peak at 134.6 mAh g^−1^. Unfortunately, the capacity retention is only 85.1% after 100 cycles. It was obvious that the optimization degree of replacing the Mn^4+^ ions with tetravalent cations cannot reach the demand for large-scale application of LiMn_2_O_4_.

It has been reported that the Cu-doping strategy can make a positive contribution in enhancing the cycling stability due to the fact that the addition of copper ions in the LiMn_2_O_4_ decrease the trivalent manganese ions and cell volume of LiMn_2_O_4_, which can inhibit the Jahn-Teller effect and enhance structural stability [23]. In this work, the LiCu_x_Mn_1.95−x_Si_0.05_O_4_ (x = 0, 0.02, 0.05, 0.08) samples have been obtained by a simple solid-state method. The effect of copper doping content on the structures, morphologies and cycling life of the LiCu_x_Mn_1.95−x_Si_0.05_O_4_ samples is discussed. The results indicate the addition of copper ions in the Si-doped spinel effectively remedy the shortcomings of the single Si-doping strategy and the Cu-Si co-doped spinel can show excellent cycling stability.

## 2. Materials and Methods 

The LiCu_x_Mn_1.95−x_Si_0.05_O_4_ (x = 0, 0.02, 0.05, 0.08) samples were synthesized by traditional high temperature solid-state reaction process using Li_2_CO_3_, electrolytic MnO_2_, C_8_H_20_O_4_Si and Cu(NO_3_)_2_ as reaction reagents. Firstly, the hydro-ball-milling technique was used to pretreat the electrolytic MnO_2_. Then, Li_2_CO_3_, electrolytic MnO_2_, Cu(NO_3_)_2_ and ethanol solution of C_8_H_20_O_4_Si were mixed thoroughly by hydro-ball-milling. The obtained slurries were dried at 70 °C and further ground into powder. Subsequently, this material was sintered at 450 °C for 4 h in air and then reground after natural cooling. The desired product LiCu_x_Mn_1.95−x_Si_0.05_O_4_ were obtained by calcining at 825 °C for 18 h in air.

The crystal structures of the obtained LiCu_x_Mn_1.95−x_Si_0.05_O_4_ samples were studied by X-ray diffraction technique (XRD, Bruker DX-1000, Karlsruhe, Germany) with Cu Kα radiation (λ = 0.15406 nm). The scanning electron microscopy (SEM, JEOL JSM-6360LV, Tokyo, Japan) analytical techniques were used to study the surface morphologies and microstructures.

The active electrode consists of the obtained LiCu_x_Mn_1.95−x_Si_0.05_O_4_ samples, conductive acetylene black and polyvinylidene fluoride (Weight Ratio = 85:10:5). The anode material and diaphragm are lithium foil and Celgard 2400 polymer, respectively. The mixture of 1 M LiPF_6_, ethyl methyl carbonate (EMC), ethylene carbonate (EC) and dimethyl carbonate (DMC) was used as electrolyte (EMC:EC:DMC = 1:1:1). The electrochemical measurement was executed on LAND (Wuhan, China) battery testing system. The electrochemical impedance spectroscopy (EIS) were tested by CS-350 electrochemical workstation (Wuhan, China). These tests were investigated by using CR2016 coin-type cells.

## 3. Results and Discussion

**Figure 1:**
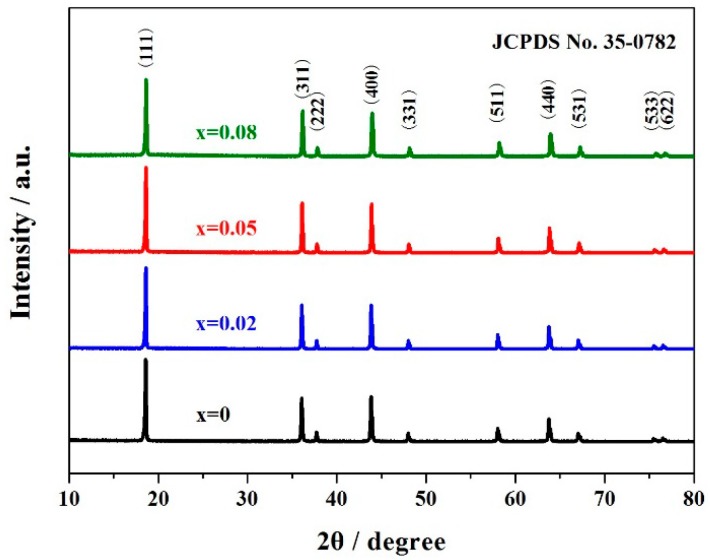
XRD patterns of LiCu_x_Mn_1.95−x_Si_0.05_O_4_ (x = 0, 0.02, 0.05, 0.08) samples.

Figure 1 shows the XRD results of the LiCu_x_Mn_1.95−x_Si_0.05_O_4_ (x = 0, 0.02, 0.05, 0.08) samples. As shown here, the characteristic peaks of all these samples match with that of LiMn_2_O_4_ (JCPDS No. 35-0782), implying that the Cu-doping strategy have no material impact on the inherent structure of LiMn_2_O_4_ [17,33], where lithium and manganese ions occupy the tetrahedral sites (8a) and octahedral sites (16d), respectively. According to the reported research result, the (220) characteristic peak may be observed if other cations occupied the tetrahedral sites [34]. However, there is no (220) characteristic peak in Figure 1, suggesting that the copper ions successfully replaced the manganese ions in octahedral sites.

According to the reported literature [35], the intensity ratio of (311)/(400) peaks can be optimized by replacing the Mn ions with some other cation ions in the spinel structure of LiMn_2_O_4_. If this intensity ratio is in the range of 0.96–1.10, the obtained samples usually show excellent cycling stability. Table 1 lists this intensity ratio of LiCu_x_Mn_1.95-x_Si_0.05_O_4_ (x = 0, 0.02, 0.05, 0.08) samples. It can be seen that the Cu-doping strategy has played a positive role in optimizing this intensity ratio. The copper and silicon co-doped spinels can present a larger intensity ratio than that of the silicon co-doped spinel. Therefore, it can be inferred that the further addition of copper ions in the silicon-doped sample may greatly enhance the cycling stability.

**Figure 2:**
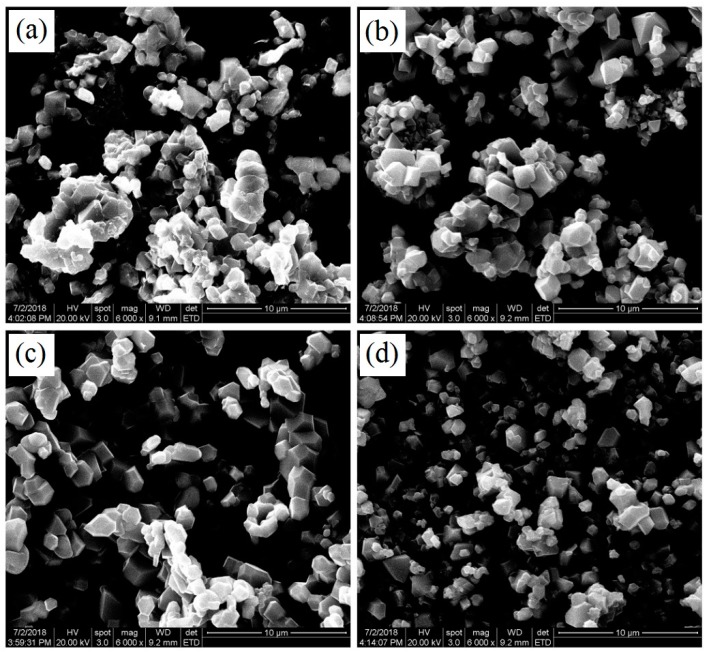
SEM images of LiCu_x_Mn_1.95-x_Si_0.05_O_4_ samples: (**a**) x = 0; (**b**) x = 0.02; (**c**) x = 0.05; (**d**) x = 0.08.

Figure 2 shows the SEM images of the LiCu_x_Mn_1.95−x_Si_0.05_O_4_ (x = 0, 0.02, 0.05, 0.08) samples. As shown in Figure 2a, the silicon-doped LiMn_2_O_4_ particles present less-than-ideal size distribution. For the copper and silicon co-doped LiMn_2_O_4_ samples, the introduction of some copper ions can further optimize the mean diameter and size distribution. When the copper doping content increases, the mean diameter of the LiCu_x_Mn_1.95−x_Si_0.05_O_4_ (x = 0.02, 0.05, 0.08) has a decreasing tendency. It is important to note that the LiCu_0.05_Mn_1.90_Si_0.05_O_4_ particles shown in Figure 2c present the quite uniform size distribution. The above-mentioned results suggest that introducing some copper ions can effectively improve the crystallinity and optimize the size distribution, which is conducive to the enhancement of cycling stability.

**Figure 3:**
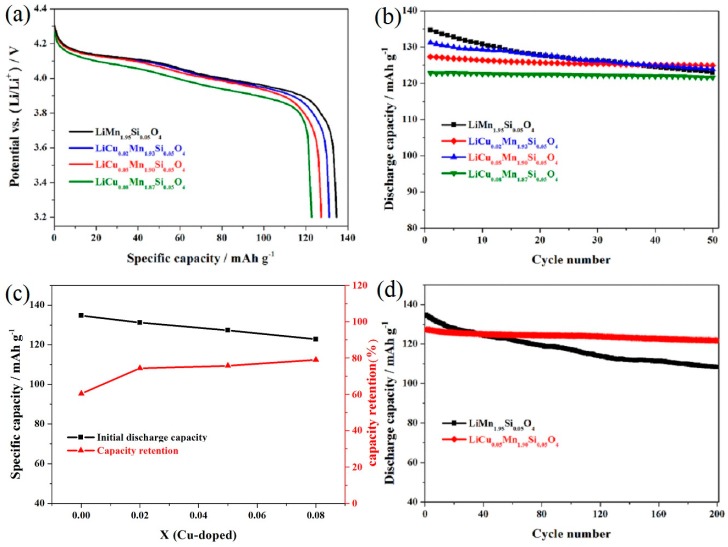
(**a**) Initial discharge curves and (**b**) Cycling performance of the LiCu_x_Mn_1.95−x_Si_0.05_O_4_ (x = 0, 0.02, 0.05, 0.08) samples; (**c**) Comparison plots of the initial discharge capacities and capacity retentions; (**d**) Long Cycling performance of the LiCu_x_Mn_1.95−x_Si_0.05_O_4_ (x = 0, 0.05) samples.

Figure 3a shows the first discharge curves of the LiCu_x_Mn_1.95−x_Si_0.05_O_4_ (x = 0, 0.02, 0.05, 0.08) samples. All these samples present characteristic discharge curves, which show two distinct voltage platforms around 4.10–4.15 V and 3.95–4.00 V, suggesting that introducing some copper ions do not change the electrochemical redox reaction mechanism and all these copper and silicon co-doped LiMn_2_O_4_ samples processes two extraction/insertion process of Li^+^ ions [14,32]. Figure 3b presents the cycling stability of the LiCu_x_Mn_1.95−x_Si_0.05_O_4_ (x = 0, 0.02, 0.05, 0.08) samples. The cycling stability of these co-doped samples were remarkably enhanced as the copper doping content increased, due to the suppressed Jahn-Teller effect and stronger structural stability [23]. Note, however, that the addition of more copper ions has a great negative impact on the reversible capacity of the LiCu_0.08_Mn_1.87_Si_0.05_O_4_ sample in spite of the improvement of cycling life (Figure 3c). These unsatisfactory results are principally because introducing more copper ions can cause the reduction of Mn^3+^, which is unfavourable to the Mn(III)–Mn(IV) interconversion.

Figure 3d shows the long cycling performance of the LiCu_x_Mn_1.95−x_Si_0.05_O_4_ (x = 0, 0.05) samples. For the LiCu_0.05_Mn_1.90_Si_0.05_O_4_ sample, the reversible capacity peaked at 127.3 mAh g^−1^, which is slightly lower than that of the LiMn_1.95_Si_0.05_O_4_ sample. After 200 cycles, the LiCu_0.05_Mn_1.90_Si_0.05_O_4_ sample can still exhibit 121.8 mAh g^−1^ with outstanding retention of 95.7%. Unfortunately, the LiMn_1.95_Si_0.05_O_4_ sample shows lower capacity with worse cycling life. After 200 cycles, this sample only delivers 108.3 mAh g^−1^ with low retention of 81.6%. According to the reference [32], the undoped LiMn_2_O_4_ only delivers a discharge capacity of 48.3 mAh g^−1^ with capacity retention of 37.8% after 100 cycles, which is much lower than that of the LiSi_0.05_Mn_1.95_O_4_ sample. Although the silicon-doping enhance the cycling performance, the further addition of copper ions can significantly enhance the cycling stability of LiMn_2_O_4_. 

**Figure 4:**
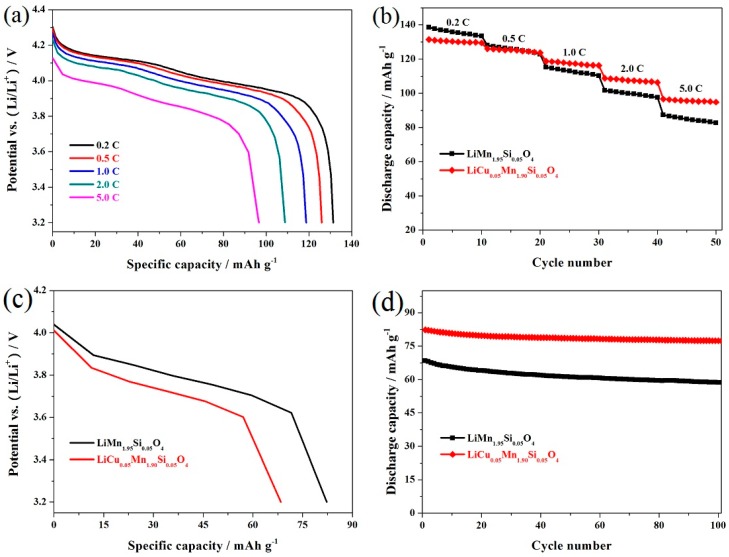
(**a**) Discharge curves of the representative LiCu_0.05_Mn_1.90_Si_0.05_O_4_ sample at varying rates; (**b**) Cycling performance of the LiCu_x_Mn_1.95−x_Si_0.05_O_4_ (x = 0, 0.05) samples at varying rates; (**c**) Initial discharge curves and (**d**) Cycling performance of the LiCu_x_Mn_1.95−x_Si_0.05_O_4_ (x = 0, 0.05) samples at 10 C.

Figure 4a shows the corresponding discharge curves of the representative LiCu_0.05_Mn_1.90_Si_0.05_O_4_ sample at varying rates. It can be seen that there are two voltage platforms which are obvious at 0.2 C and 0.5 C, suggesting the diffusion process of lithium ions [36]. When the rate further increases, these two potential plateaus gradually show ambiguous boundary and shifted toward lower voltage. This result has a lot to do with the polarization effect and ohmic drop [37]. Figure 4b shows the cycling stability of the LiCu_0.05_Mn_1.90_Si_0.05_O_4_ and LiMn_1.95_Si_0.05_O_4_ samples at varying rates. When cycled at 0.2 C, the capacities of these two samples reached up to 138.5 and 131.4 mAh g^−1^, respectively. However, what is important to pay attention to is the reversible capacity of the LiCu_0.05_Mn_1.90_Si_0.05_O_4_ sample, which showed much more obvious difference at high rates of 5.0 C.

To further study the cycling performance at a high rate, the LiCu_0.05_Mn_1.90_Si_0.05_O_4_ and LiMn_1.95_Si_0.05_O_4_ samples were tested at 10 C. For the LiCu_0.05_Mn_1.90_Si_0.05_O_4_ sample, the two characteristic voltage plateaus shown in Figure 4c become blurred to a certain extent. By contrast, the LiMn_1.95_Si_0.05_O_4_ presents lower voltage plateau and corresponding to this, the capacity of this material shows severe degradation. Figure 4d presents the cycling life of these two spinels at 10 C. The LiMn_1.95_Si_0.05_O_4_ sample shows unsatisfactory capacity retention of 85.7% with low initial capacity of 68.4 mAh g^−1^, while the LiCu_0.05_Mn_1.90_Si_0.05_O_4_ sample can display a higher capacity of 82.3 mAh g^−1^. More importantly, after 100 cycles, the corresponding retention can reach up to 94.0% with the 100th cycle with a capacity of 77.4 mAh g^−1^. The above discussion indicates that the introduction of copper ions has great value in the optimization of the rate capability.

**Figure 5:**
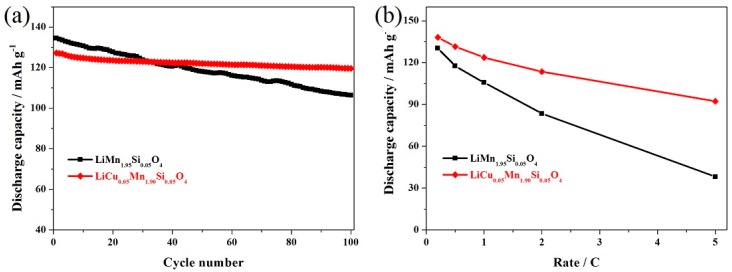
(**a**) Cycling performance and (**b**) rate capacities of the LiCu_x_Mn_1.95−x_Si_0.05_O_4_ (x = 0, 0.05) samples at 55 °C.

Figure 5 shows the electrochemical properties of the LiCu_0.05_Mn_1.90_Si_0.05_O_4_ and LiMn_1.95_Si_0.05_O_4_ samples at 55 °C. As shown in Figure 5a, the LiCu_0.05_Mn_1.90_Si_0.05_O_4_ exhibits an initial capacity of 127.2 mAh g^−1^ at 0.5 C. After 100 cycles, this sample still maintains a high capacity of 119.6 mAh g^−1^ with excellent retention of 94.0%. However, the LiMn_1.95_Si_0.05_O_4_ sample shows much lower retention than that of the LiCu_0.05_Mn_1.90_Si_0.05_O_4_. The capacity retention of the LiMn_1.95_Si_0.05_O_4_ sample is only 79.1% with a lower capacity of 106.4 mAh g^−1^ after 100th cycle. Such low discharge capacity after 100 cycles is mostly given to the fact that the high temperature accelerates the dissolution of manganese and undermines the structural stability of LiMn_2_O_4_. Note, however, that the LiCu_0.05_Mn_1.90_Si_0.05_O_4_ sample can still show much better cycling stability although these two samples show low discharge capacity after 100 cycles. These results suggest that introducing some copper ions can be favorable for enhancing the cycling stability at high-temperature. Figure 5b shows the rate capability of these two samples at 55 °C. When cycled at low rates, the LiCu_0.05_Mn_1.90_Si_0.05_O_4_ and LiMn_1.95_Si_0.05_O_4_ samples show similar capacities. However, as the cycling rate increased, these two samples gradually show some difference. When cycled at 5.0 C, the LiCu_0.05_Mn_1.90_Si_0.05_O_4_ sample can show 103.4 mAh g^−1^ while the LiMn_1.95_Si_0.05_O_4_ only shows 91.7 mAh g^−1^. The above-mentioned results suggest that the addition of copper ions can further improve the rate capability of LiMn_1.95_Si_0.05_O_4_ at high-temperature.

**Figure 6:**
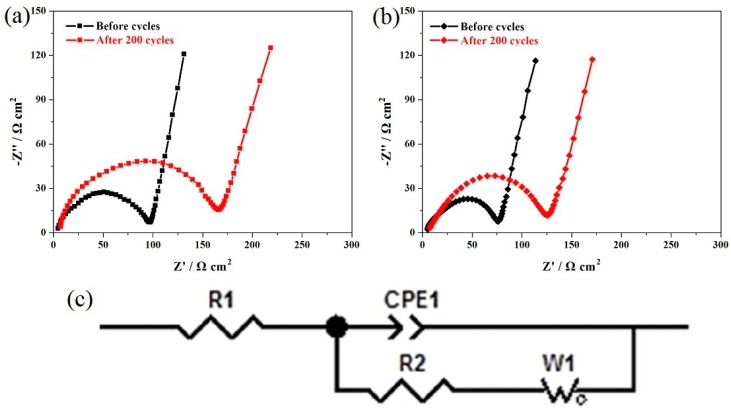
Nyquist plots of the LiMn_1.95_Si_0.05_O_4_ (**a**) and LiCu_0.05_Mn_1.90_Si_0.05_O_4_ (**b**) samples before cycling and after 200 cycles; (**c**) Equivalent circuit model of EIS.

Figure 6a,b show the EIS results of the LiCu_0.05_Mn_1.90_Si_0.05_O_4_ and LiMn_1.95_Si_0.05_O_4_ samples. It has been reported previously that the charge transfer resistance (R_2_) corresponds to the high-frequency semicircle, which has a connection with the cycling life [14,34]. Therefore, we mainly determine the R_2_ values to confirm the effect of introducing copper ions on the cycling stability. Table 2 lists the fitting values of R_2_. For the LiCu_0.05_Mn_1.90_Si_0.05_O_4_ sample, the original R_2_ value only reach 70.2 Ω cm^2^ but increase to 116.0 Ω cm^2^ after 200 cycles. The R_2_ value increase was relatively small with low growth rate of 64.5%. By contrast, the LiMn_1.95_Si_0.05_O_4_ shows a higher original R_2_ value (93.2 Ω cm^2^). However, this value increases to 158.1 Ω cm^2^ with growth rate of 69.6%. Through the above analysis, it is concluded that replacing some trivalent manganese ions with copper ions can have a constructive role in decreasing the R_2_ value and enhancing the diffusion of lithium ions [33,38,39].

## 4. Conclusions

The LiCu_x_Mn_1.95−x_Si_0.05_O_4_ (x = 0, 0.02, 0.05, 0.08) samples have been obtained by a simple solid-state method. The further addition of copper ions in the LiMn_2_O_4_ can decrease the trivalent manganese ions and cell volume of LiMn_2_O_4_, which can inhibit the Jahn-Teller effect and enhance structural stability. As the optimal Cu-Si co-doped spinel, the LiCu_0.05_Mn_1.90_Si_0.05_O_4_ sample possessed even size distribution. More importantly, it showed much better cycling stability and elevated temperature performance than the Si-doped LiMn_2_O_4_ sample. When cycled at 0.5 C, the LiCu_0.05_Mn_1.90_Si_0.05_O_4_ sample exhibited 127.3 mAh g^−1^, which is slightly lower than that of the LiMn_1.95_Si_0.05_O_4_ sample. After 200 cycles, the LiCu_0.05_Mn_1.90_Si_0.05_O_4_ sample could exhibit 121.8 mAh g^−1^ with outstanding retention of 95.7% at 0.5 C. Moreover, this co-doped sample can show outstanding rate capability and high-temperature performance. All these results suggest that the further addition of copper ions can produce an obvious effect in enhancing the cycling stability of the silicon-doped LiMn_2_O_4_.

## Figures and Tables

**Table 1:** Intensity ratio of (311)/(400) peaks of LiCu_x_Mn_1.95−x_Si_0.05_O_4_ (x = 0, 0.02, 0.05, 0.08) samples.

Samples	I_(311)_/I_(400)_
LiMn_1.95_Si_0.05_O_4_	0.98
LiCu_0.02_Mn_1.93_Si_0.05_O_4_	1.00
LiCu_0.05_Mn_1.90_Si_0.05_O_4_	1.01
LiCu_0.08_Mn_1.87_Si_0.05_O_4_	1.03

**Table 2:** Fitting values of the charge transfer resistance (R_2_) calculated from EIS.

Samples	R_2_ (Ω cm^2^) before Cycles	R_2_ (Ω cm^2^) after 200 Cycles
LiMn_1.95_Si_0.05_O_4_	93.2	158.1
LiCu_0.05_Mn_1.90_Si_0.05_O_4_	70.5	116.0
